# Research Progress on the Interaction Between Autophagy and Energy Homeostasis in Cardiac Remodeling

**DOI:** 10.3389/fphar.2020.587438

**Published:** 2020-11-30

**Authors:** Wen Ding, Hong Feng, Wen-jing Li, Hai-han Liao, Qi-zhu Tang

**Affiliations:** ^1^ Department of Cardiology, Renmin Hospital of Wuhan University, Wuhan, China; ^2^ Hubei Key Laboratory of Metabolic and Chronic Diseases, Wuhan, China; ^3^ Department of Geriatrics, Renmin Hospital of Wuhan University, Wuhan, China

**Keywords:** autophagy, energy homeostasis, cardiac remodeling, cardiac hypertrophy, diabetes-associated cardiomyopathy, ischemic heart diseases

## Abstract

Cardiac remodeling is a common pathological process in various heart diseases, such as cardiac hypertrophy, diabetes-associated cardiomyopathy and ischemic heart diseases. The inhibition of cardiac remodeling has been suggested to be a potential strategy for preventing heart failure. However, the mechanisms involved in cardiac remodeling are quite complicated. Recent studies have reported a close correlation between autophagy and energy homeostasis in cardiac remodeling associated with various heart diseases. In this review, we summarize the roles of autophagy and energy homeostasis in cardiac remodeling and discuss the relationship between these two processes in different conditions to identify potential targets and strategies for treating cardiac remodeling by regulating autophagy.

## Introduction

Cardiac remodeling refers to a series of changes in left ventricular function and structure caused by various cardiovascular diseases or pathological risk factors. Cardiac remodeling leads to fibrosis, ventricular dilatation and cardiac hypertrophy, all of which will inevitably impair the cardiac systolic and diastolic function and ultimately progress into heart failure (Wu et al., 2017). Although researchers have focused on illuminating the potential mechanisms of malignant cardiac remodeling and heart failure, some problems related to the mechanisms of cardiac remodeling remained to be clarified, which limits the further development and progress in the identification of new and effective therapies for preventing or even reversing cardiac remodeling (Tham et al., 2015).

The process of cardiac remodeling is often accompanied by the activation of inflammation, enhanced myocardial fibrosis, abnormal autophagy and disordered metabolism. Studies have documented the critical roles of autophagy and energy homeostasis in cardiac remodeling and the close relationship between these processes (Bertero and Maack, 2018). Nutrient deficiency caused by insufficient energy metabolism may initiate autophagy to degrade energy storage substrates, including proteins, fat and glycogen, to meet the energy requirements of cells (Gustafsson and Gottlieb, 2008; Vigetti et al., 2018). In turn, the accumulation of nutrients generated by autolysosomes may activate mammalian target of rapamycin complex 1 (mTORC1) to terminate autophagy (Tan and Miyamoto, 2016). This feedback avoids excess activation of autophagy. Therefore, autophagy plays an important regulatory role in maintaining energy homeostasis. An autophagy disorder will lead to abnormal energy metabolism, while the excess nutrients caused by the energy metabolism disorder will also affect autophagy (Galluzzi et al., 2014). Thus, the autophagy-energy system must be coordinated to maintain the dynamic balance of the physiological state of cells and organisms. An understanding of the accurate mechanisms underlying the association between autophagy and energy homeostasis is very important to prevent or even reverse the progression of pathological cardiac remodeling.

## Autophagy in Cardiac Remodeling

Autophagy is a progress of cellular self-cannibalism that degrades damaged organelles, misfolded proteins and other macromolecular substances required for the energy supply by transporting these substrate to lysosomes and forming the autophagosome (Delbridge et al., 2017; Dikic and Elazar, 2018). According to the method in which substances are delivered to lysosomes, autophagy is divided into three categories: macroautophagy, microautophagy, and chaperone-mediated autophagy (Delbridge et al., 2017). Among these categories, the most common and well-studied type of autophagy is macroautophagy. Macroautophagy is usually referred as autophagy. Autophagy has long been thought to be nonspecific. However, recent studies show that autophagy also targets at specific organelles and proteins, which is termed as specific autophagy (Anding and Baehrecke, 2017), and represents an important mechanism to remove damaged mitochondria in cells. Mitophagy is one of the most well-studied forms of specific autophagy and is an indispensable part of organized mitochondrial quality control and cardiac energy homeostasis (Dorn, 2019). Compromised mitophagy may lead to mitochondrial dysfunction and lipid accumulation in individuals with diabetes-associated cardiomyopathy (Tong et al., 2019). The improvement in mitophagy by melatonin administration might protect mitochondrial energy metabolism from myocardial ischemia-reperfusion injury (Zhang et al., 2019). Mitophagy plays a role in autophagy and energy homeostasis during cardiac remodeling.

Autophagy is an important regulator of cardiac homeostasis and function under normal conditions. For example, starvation-induced autophagy maintains left ventricular function by increasing the myocardial ATP and amino acid contents (Takemura et al., 2009; Hariharan et al., 2010), exercise-induced autophagy promotes cardiac glucose metabolism by increasing glucose uptake (He et al., 2012), and aging-associated autophagy prevents aging-related cardiac dysfunction by clearing misfolded proteins and dysfunctional mitochondria (Miyamoto, 2019). Under stress such as starvation and exercise, an ATP deficiency and increased AMP/ATP ratio activate AMP-activated protein kinase (AMPK) in cardiomyocytes (Kemp et al., 1999). Activated AMPK enhances autophagy by inhibiting mammalian target of rapamycin (mTOR) or phosphorylating unc-51 such as kinase-1 (ULK1), which induce ATP production (Matsui et al., 2007; Gustafsson and Gottlieb, 2009; Qi and Young, 2015). Consequently, increased autophagy provides supplemental substrates for energy metabolism, supplies amino acids for protein synthesis, and clears damaged mitochondria, all of which may limit cardiomyocyte death (Sciarretta et al., 2018a).

In contrast, physiological conditions, the role of autophagy in pathological conditions is complicated and varies in specific contexts. In the infarcted mouse heart, microtubule-associated protein light chain 3 (LC3) I, LC3II and sequestosome 1 (p62/SQSTM1) were significantly upregulated both at first and third weeks, respectively, in surviving cardiomyocytes (Wang et al., 2018). Autophagosome biogenesis requires LC3-mediated elongation to form a circular double-layer membrane. LC3 I is distributed in the cytoplasm, while LC3II is located in autophagosomes. Therefore, the transition from LC3 I to LC3II indicates the increased formation of autophagosomes (Florey and Overholtzer, 2012; Hamacher-Brady, 2012). p62 is an autophagy receptor that interacts with phagophores with its LC3 domain and interacting with the ubiquitin-proteasome system via its ubiquitin-associated domain to mediate autophagosome degradation (Johansen and Lamark, 2011). The accumulation of p62 indicates a blockade ofautophagy flux. Thus, p62 and LC3 are both important markers of autophagy and are often used to monitor changes in autophagy. Treatment with autophagy inhibitors significantly aggravates cardiac dysfunction and cardiac remodeling after infarction (Kanamori et al., 2011). Moreover, the activation of autophagy by mTOR inhibitors or other autophagy enhancers may reduce the area of the myocardial infarct and mitigate left ventricular remodeling after myocardial infarction (Buss et al., 2009; Sciarretta et al., 2018b). Therefore, autophagy activation during myocardial infarction is an adaptive response to protect the heart from injury, and inadequate upregulation of autophagy may lead to heart failure after myocardial infarction.

However, autophagy appears to play a completely opposite role in myocardial ischemia-reperfusion. As shown in the study by Ma et al. (2012), myocardial ischemia-reperfusion impaired autophagosome clearance and increased cardiomyocyte death, partially due to the reduced level of lysosome-associated membrane protein-2 but up-regulated Beclin-1 expression induced by reactive oxygen species. Additionally, autosis, a novel form of cell death induced by autophagy, was observed in myocardial ischemia-reperfusion. The suppression of autosis reduced cardiac injury, which further confirmed the adverse effects of autophagy on myocardial ischemia-reperfusion (Nah et al., 2020). Moreover, the inhibition of autophagy by either a histone deacetylase inhibitor or autophagy-related circular RNA (ACR) reduces the infarct area and attenuates myocardial ischemia-reperfusion injury (Xie et al., 2014; Zhou et al., 2019). The opposite effects of autophagy on myocardial infarction and myocardial ischemia-reperfusion may be attributed to the finding that autophagy in the first process is mediated by AMPK but mediated by Beclin1 in the second process (Matsui et al., 2007; Takagi et al., 2007; Shi et al., 2019).

Mice with a cardiac-specific deficiency in autophagy-related 5 showed a disorderly arrangement of the cardiac sarcomere structure, an abnormal mitochondrial structure and obvious cardiac dysfunction after the induction of aortic constriction-induced pressure overload (Nakai et al., 2007). However, overexpression of Beclin1 aggravated the pathological cardiac remodeling under pressure overload (Zhu et al., 2007). Based on these studies, baseline autophagy is necessary for maintaining the cardiac structure and function, but overactivation by beclin1 overexpression might be detrimental. In diabetic mice, the inhibition of AMPK activity reduced cardiac autophagy, which aggravated cardiac dysfunction and increased mortality in diabetic mice (Zou and Xie, 2013). Xie Z et alused metformin to chronically activate AMPK and prevent a deterioration in cardiac function via increasing autophagy activity (Xie et al., 2011a; Xie et al., 2011b). In contrast, Chen et al. (2017) inhibited Beclin1 expression with Mir30c and observed an amelioration of damage to the cardiac structure and function in diabetic mice. Therefore, autophagy plays a dual role in cardiac remodeling. Autophagy is activated to different levels and exerts quite different effects on different animal models, ultimately producing beneficial or harmful effects. In view of the complexity of autophagy, artificial activation or inhibition of a specific target may fail to imitate changes in autophagy flux itself. Moderate autophagy activity will be beneficial for reducing intracellular aging organelles and providing energy metabolism substrates for cells in ischemia, while excessively activated autophagy may selectively degrade some key metabolic enzymes and mitochondria, leading to metabolic disorders that accelerate the process of cardiac decompensation and ultimately result in heart failure. The protective effect of autophagy on the heart is only achieved when autophagy is maintained at the appropriate level.

## Energy Homeostasis in Cardiac Remodeling

The heart expends a large amount of energy to function normally and pump blood with frequent contractions. The ATP turnover rate in the human heart is 15–20 times its own weight (Kolwicz et al., 2013). Since the heart has little reserve of high-energy phosphate, a sustainable and stabile ATP supply is essential for the maintenance of cardiac systolic function. The energy metabolism of the heart is flexible to ensure an adequate ATP supply in response to different pathophysiological stresses (Bertero and Maack, 2018). Various energy substrates, such as carbohydrates, lipids, amino acids and ketone bodies, can be utilized for the different the biological activities of cardiomyocytes (Stanley et al., 2005). Although many different substrates supply energy to cardiomyocytes, fatty acid oxidation is the main source of the energy supply in the adult heart, accounting for approximately 70–90% of the total energy consumption in the adult heart, and the remaining 10–30% is derived from the oxidation of glucose and lactate (Lopaschuk et al., 2010; Doenst et al., 2013).

Cardiac remodeling is often accompanied by the remodeling of energy metabolism. In pressure overload-induced cardiac remodeling, glycolysis is enhanced and fatty acids oxidation is reduced (Krishnan et al., 2009), which has been observed at 2 weeks after aortic constriction in rats (Doenst et al., 2010). Increased glucose utilization in cardiac hypertrophy promotes aspartate biosynthesis, leading to the increased synthesis of nucleotides, RNA and proteins (Nakamura and Sadoshima, 2018). A deletion of acetyl-CoA-carboxylase2 (ACC2) increases fatty acid oxidation to maintain catabolic metabolism for energy production and avoid anabolic metabolism (Ritterhoff et al., 2020). Indeed, the energy metabolism pattern in cardiac pressure overload-induced cardiac remodeling is similar to the fetal heart, which is characterized by reduced ATP synthesis that leads to inefficient energy metabolism (Sorokina et al., 2007). In contrast to energy metabolism in cardiac hypertrophy, the energy metabolism in diabetes and obesity-induced cardiac remodeling is characterized by increased fatty acid intake and oxidation but decreased glucose oxidation because of impaired insulin signaling (Anderson et al., 2009). As a result, the oxygen consumption of the heart is increased, but the efficiency of energy metabolism is decreased and accompanied by the production of excess oxidative stress, which contributes to the imbalance between an increased substrate supply and decreased oxidative phosphorylation capacity (Boudina et al., 2007; Anderson et al., 2009; Kolwicz et al., 2013). Although energy metabolic remodeling is varies in different cardiac remodeling models, the development of heart failure is accelerated by common factors, including oxidative stress caused by continuous metabolic disorders, increased insulin resistance, lipid accumulation and energy deficiency (Stanley et al., 2005; Ashrafian et al., 2007; Ingwall, 2009; Tuunanen and Knuuti, 2011; Noordali et al., 2018).

## Interaction Between Autophagy and Energy Homeostasis in Cardiac Hypertrophy

Cardiac hypertrophy is an adaptation of the heart that increases cardiac contraction and decreases ventricular wall stress in response to hemodynamic overload. Cardiac hypertrophy is divided into physiological hypertrophy and pathological hypertrophy (Shimizu and Minamino, 2016; Nakamura and Sadoshima, 2018). Physiological cardiac hypertrophy is reversible and not accompanied by cardiac dysfunction. During physiological cardiac hypertrophy, autophagy is strictly controlled at the basal level and energy efficiency is improved, while autophagy is up-regulated and metabolism is reprogrammed during pathological cardiac hypertrophy (Rothermel and Hill, 2008; Nakamura and Sadoshima, 2018). As a result, the abnormal metabolism and autophagy caused by pathological signaling pathways will cause cardiac dysfunction and eventually contribute to heart failure. The subsequently occurring cardiac hypertrophy is pathological cardiac hypertrophy.

**FIGURE 1:**
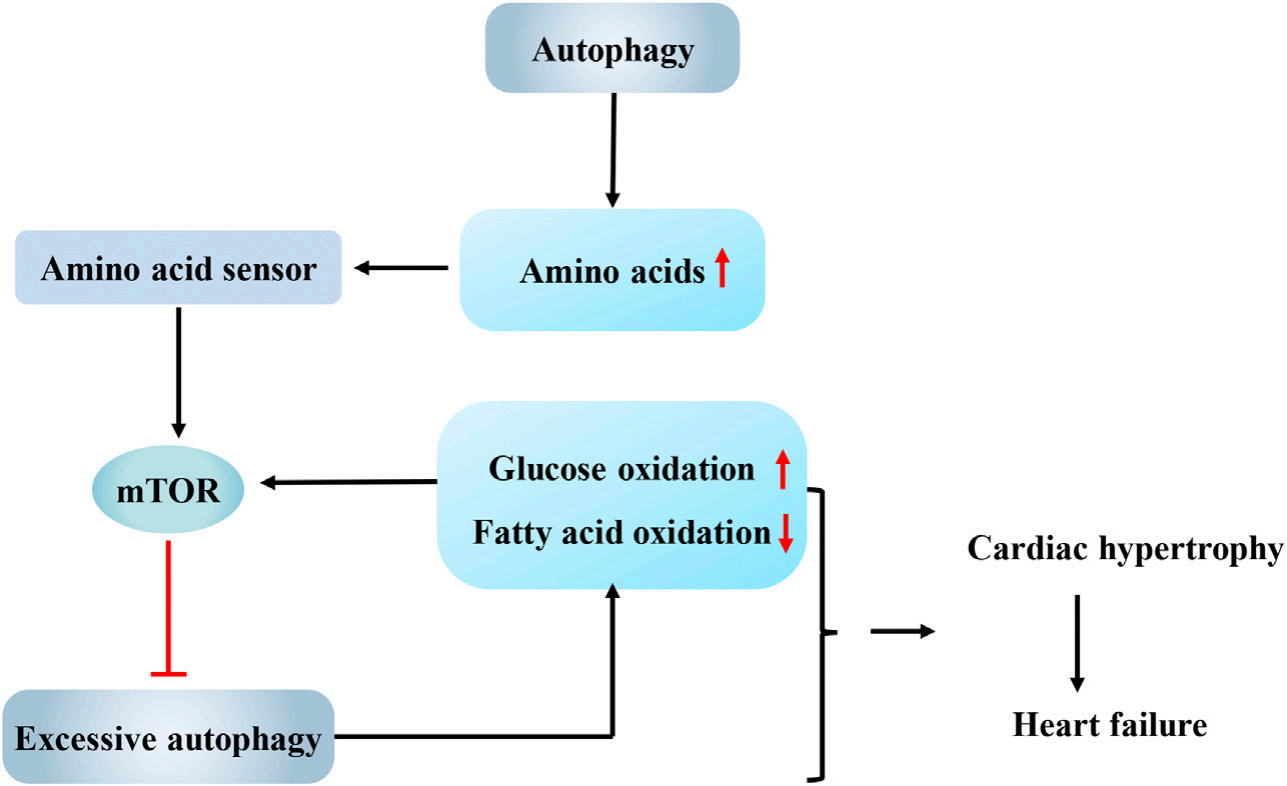
Interaction between autophagy and energy metabolism in cardiac hypertrophy. Autophagy supplies amino acids for protein synthesis in cardiac hypertrophy, while abundant amino acids interact with amino acid sensors to activate mammalian target of rapamycin (mTOR) and avoid excess autophagy activation. Overactivated autophagy leads to a shift in energy metabolism from fatty acid oxidation to glucose oxidation. In turn, the change in the energy substrate can activate mTOR and then inhibit autophagy. And the interaction between them may be one explanation for the observation that cardiac hypertrophy eventually progresses to heart failure. mTOR, mammalian target of rapamycin.

In endothelin-1-treated cardiomyocytes, prolyl-tRNA synthase inhibitors activate the amino acid response, which mimics amino acid deprivation to enhance autophagy flux, as evidenced by increased LC3II expression and decreased p62 accumulation. Autophagy mediated by the amino acid response maintains the metabolic balance (Qin et al., 2017). When amino acids are abundant, Rag GTPases, which function as amino acid sensors, activate mTORC1 to inhibit autophagy by recruiting mTORC1 onto the lysosomal membrane surface (Jewell et al., 2013; Bar-Peled and Sabatini, 2014). Mice with cardiomyocyte-specific RagA and RagB knockout showed abnormal deposition of autophagosomes and autolysosomes and malignant cardiac remodeling after challenge with pressure overload (Kim et al., 2014). Similarly, knockout of the G protein coupled receptor TAS1R3, another amino acid sensor, also enhanced autophagy in the mouse heart (Wauson et al., 2012). The authors of these studies concluded that amino acids are important regulators of cardiac autophagy. When the energy supply is sufficient, amino acids interact with amino acid sensors to activate mTORC1 and avoid excess autophagy activation (Figure 1). When protein metabolism is blocked, the amino acid response in cardiomyocytes will activate autophagy, which helps the heart response to the stimulation by pro-cardiac hypertrophy factors. In addition, the maintenance of high levels of acetyl-CoA inhibits excess autophagy in cardiac hypertrophy caused by pressure overload (Marino et al., 2014). Because acetyl-CoA is the common pathway through which glucose, lipids and proteins, the three major metabolisms, enter the Krebs cycle, sufficient energy metabolism might be an important prerequisite for limiting excess autophagy in cardiac hypertrophy.

Raptor is an important component of mTORC1. According to Shende et al. (2011), autophagy is significantly enhanced in the mouse heart after raptor knockout. The raptor knockout mice developed acute dilated cardiomyopathy accompanied by reduced palmitate oxidation and increased glucose oxidation after challenge with pressure overload, indicating a shift in energy metabolism from fatty acid oxidation to glucose oxidation. Based on the results of this study, the overactivation of autophagy not only inhibits the overload-induced adaptive hypertrophy of the heart but also increases glucose oxidation, shifting the main substrate of energy metabolism from fatty acids to glucose. Quite a few studies observed this pathological shift in energy metabolism in the decompensated stage of heart failure (Stanley et al., 2005; Bedi et al., 2016; Gibb and Hill, 2018), and this shift was proven to aggravate myocardial lipotoxicity and accelerate cardiac decompensation (Tuunanen et al., 2006; Santos-Gallego et al., 2019). Fatty acids are converted to acyl-CoAs by acyl-CoA synthetases (ACSLs), which are then oxidized in the mitochondria to produce energy. As shown in the study by Grevengoed et al. (2015), temporary knockout of acyl-CoA synthetase 1 (Acsl1^T−/−^) increased glucose oxidation by eight-fold in the mouse heart to compensate for the compromised fatty acid oxidation. The change in the energy substrate activated mTORC1 and then inhibited autophagy, ultimately resulting in a three-fold increase in the number of structurally abnormal mitochondria in the heart. Rapamycin-induced autophagy activation clears abnormal mitochondria and normalizes mitochondrial function in Acsl1^T−/−^ hearts. Therefore, although the increased glucose utilization rate in the early stage of cardiac hypertrophy might meet the needs of growing cardiomyocytes as an adaptive mechanism, when the fatty acid oxidation is almost completely replaced by glucose oxidation, the inhibition of autophagy will fail to clear damaged mitochondria and ultimately affects the synthesis of ATP, which may be one explanation for the observation that cardiac hypertrophy eventually progresses to heart failure (Figure 1).

Obviously, as a sensor for the change in the energy metabolic substrate and an inhibitor of autophagy in cardiac hypertrophy, mTOR is an important target for treating cardiac hypertrophy. Therapeutic interventions such as resveratrol and metformin effectively attenuate cardiac hypertrophy by activating AMPK and inhibiting the mTOR pathway (Dolinsky et al., 2009; Fu et al., 2011). Therefore, mTOR may be the key to exploring the interaction between autophagy and energy homeostasis, and methods to simultaneously optimize autophagy and energy homeostasis by regulating mTOR in certain contexts may become a challenge in future studies.

## Interaction Between Autophagy and Energy Homeostasis in Diabetes-Associated Cardiomyopathy

**FIGURE 2:**
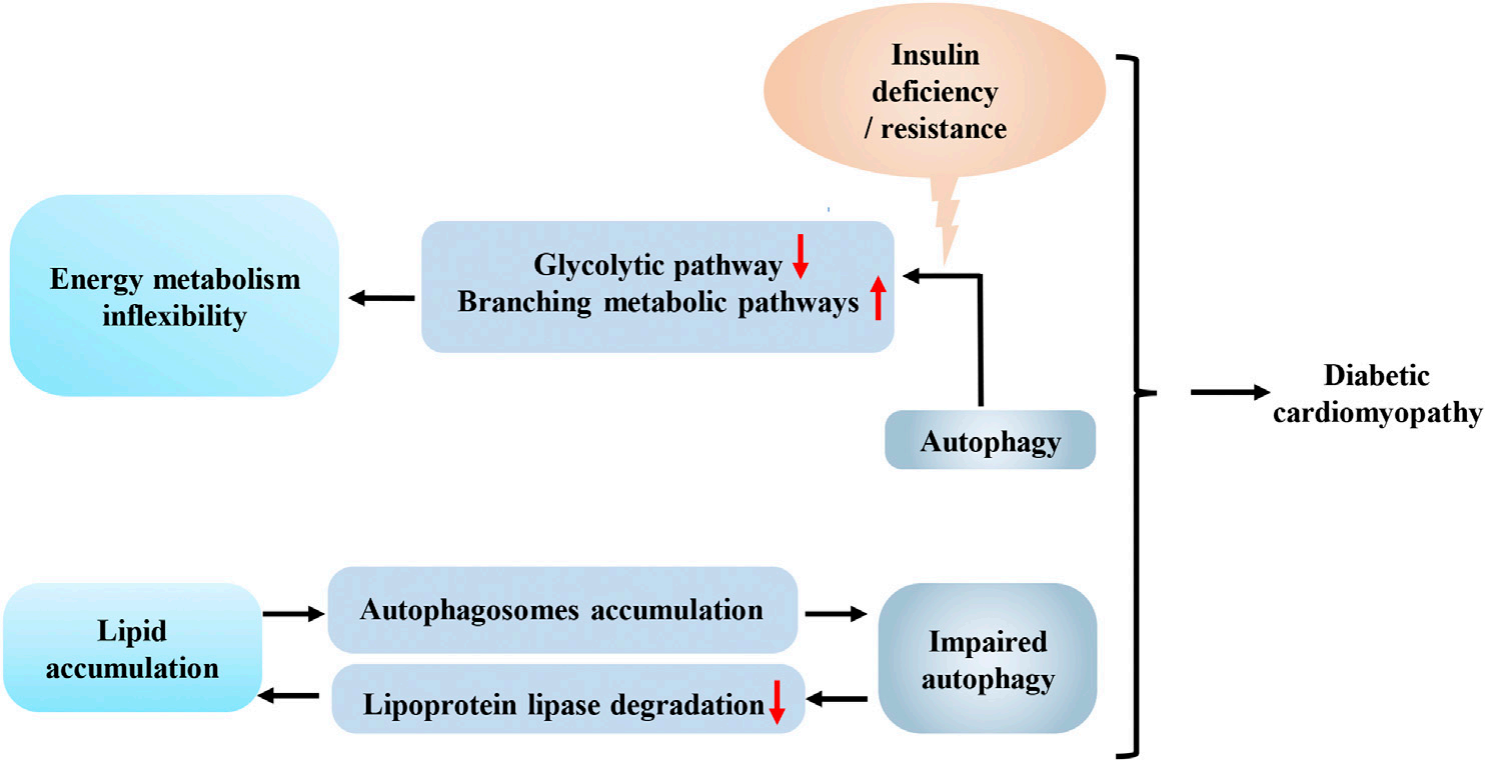
Interaction between autophagy and energy metabolism in diabetes-associated cardiomyopathy. Decreased insulin signal transduction in the heart shifts glucose from glycolytic pathway to branching metabolic pathways by autophagy, which reduces the efficiency of energy metabolism and increases the cardiac the inflexibility of cardiac metabolism. Increased lipid deposition can lead to impaired autophagy, which in turn aggravates the accumulation of lipid in the heart. This vicious circle, combined with the inflexibility of energy metabolism, exacerbates diabetes-associated cardiomyopathy.

Diabetes-associated cardiomyopathy refers to the structural and functional changes in the heart caused by diabetes in the absence of coronary artery disease, hypertension, valvular heart disease or other cardiovascular diseases (Tan et al., 2020). In mice with type 2 diabetes mellitus (T2DM), increased fatty acid levels downregulate SP1 expression. SP1 is an important transcription factor involved in energy metabolism. SP1 downregulation relieves the inhibition of the downstream Mir30c and results in BECN1 overexpression, which facilitates the formation of autophagosomes in the initial phase of autophagy. Increased autophagy induces cell death and finally exacerbates cardiac remodeling and fibrosis in diabetes-associated cardiomyopathy (Chen et al., 2017). Additionally, in the hearts of high-fat diet-fed mice and palmitate-stimulated H9C2 cardiomyocytes, excess fatty acids impair lysosomal enzyme activity by activating the protein kinase C-β (PKCβ)/NADPH oxidase 2 (NOX2) pathway, which blocks the digestion of autophagosome, resulting in decreased autophagy flux and the accumulation of autophagosomes (Jaishy et al., 2015). According to An et al., a ULK1 deficiency prevented autophagy-mediated lipoprotein lipase (LPL) degradation in the hearts of high-fat diet-fed mice and resulted in the excess deposition of fatty acids in cardiomyocytes, which caused cardiac dysfunction (An et al., 2017). Tong et al. reported that an autophagy-related 7 (ATG7) deletion impaired mitochondrial function and increased lipid deposition in the hearts of mice fed a high-fat diet (Tong et al., 2019). Based on these studies, we concluded that autophagy plays important roles in diabetes-associated cardiomyopathy. Abnormally increased fatty acid levels initiate autophagy, but accumulated fatty acids impair the digestion and degradation of autophagosomes, resulting in autophagosome accumulation. The accumulated autophagosomes may be one explanation for the aggravated cardiac remodeling in individuals with T2DM (Figure 2).

Although both type 1 diabetes mellitus (T1DM) and T2DM are characterized by similar metabolic disorders, such as hyperglycemia and dyslipidemia, the characteristics of autophagy in the heart of individuals with T1DM is somewhat different from the heart of individuals with T2DM. Kanamori H et al. (Kanamori et al., 2015) observed an increase in the whole process of autophagy in the hearts of individuals with type 1 diabetes, while the final digestion step of autophagy was suppressed in the hearts of individuals with type 2 diabetes. Zhang et al. (2016) confirmed that fenofibrate, a lipid-lowering drug, prevents fibrosis and inflammation in the hearts of subjects with type 1 diabetes upregulating autophagy mediated by sirtuin1. Wei et al. (2017) also reported that enhancing autophagy by inhibiting mTOR in type 1 diabetic rats restrained the development of diabetes-associated cardiomyopathy. Thus, enhanced autophagy might alleviate T1DM-associated cardiomyopathy. Nevertheless, the exact effect of lipid metabolism on autophagy in T1DM-related cardiomyopathy still requires further studies. Furthermore, since the suppression of the digestion step of autophagy in the hearts of individuals with type 2 diabetes is potentially explained by the effects of lipid metabolism, a fascinating problem that remains to be explored is whether the differences in autophagy characteristics in the hearts of individuals with T2DM and T1DM are explained by the effects of energy metabolism.

Insulin deficiency in individuals with T1DM and insulin resistance in individuals with T2DM may lead to decreased insulin signal transduction in the heart, which facilitates the degradation of phosphofructokinase 2 (PFK-2) by chaperone-mediated autophagy. Meanwhile, phosphofructokinase 1 (PFK-1), the rate-limiting enzyme in the glycolytic pathway, is also inhibited due to the degradation of PFK-2 (Bockus et al., 2017). Then, the excess accumulation of upstream intermediates shifts glucose from the glycolytic pathway to other energy metabolism pathways, such as glycogen synthesis and the pentose phosphate pathway, and this shift will reduce the efficiency of energy metabolism and increase the inflexibility of cardiac metabolism, which is one of the common pathological mechanisms of diabetes-associated cardiomyopathy associated with T1DM and T2DM (Figure 2). In other words, the interaction between autophagy and energy homeostasis amplifies the adverse effects of decreased insulin signal transduction caused by insulin deficiency or resistance in the hearts of individuals with diabetes. The reduced insulin signaling results in increased lipid deposition and less glucose utilization, and autophagy subsequently enhances the aforementioned adverse effects. Further studies of this relationship may facilitate the development of new strategies for the treatment of diabetes-associated cardiomyopathy.

## Interaction Between Autophagy and Energy Homeostasis in Ischemic Heart Diseases

**FIGURE 3:**
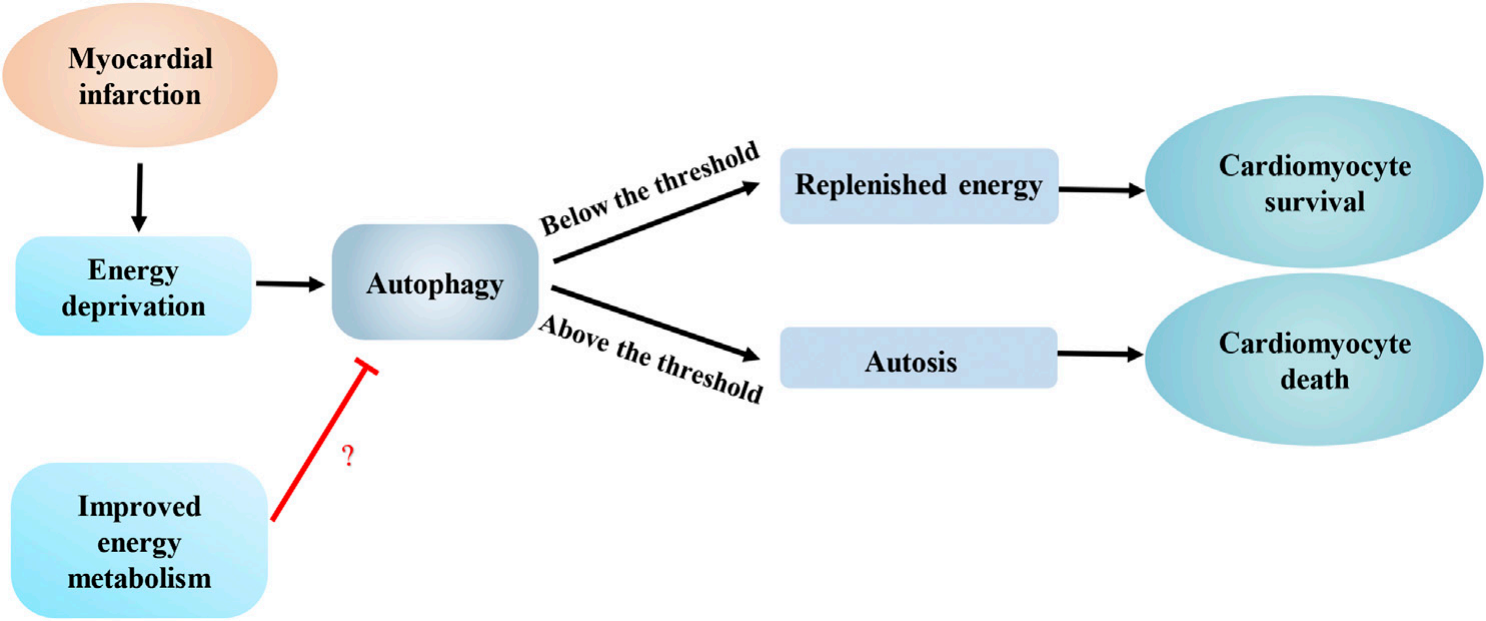
Interaction between autophagy and energy metabolism in ischemic heart diseases. Energy deprivation caused by myocardial infarction enhances autophagy to promote cardiomyocyte survival by replenishing energy, however, excess autophagy can induce autosis, which can lead to cardiomyocyte death. An improvement in energy metabolism melay limit autophagy to an appropriate level.

During myocardial infarction, cardiomyocytes are in an energy-deficient status. Accordingly, AMPK-mediated autophagy plays an important role in protecting against cardiomyocyte injuries by supplying energy metabolism substrates. An injection of rapamycin reduces the myocardial infarct area of mice fed a high-fat diet (Sciarretta et al., 2012). Hexokinase- II (HK-II), a key enzyme in the glycolytic pathway, interacts with mTORC1 and inhibits its activity to enhance autophagy. Glucose-6-phosphate, which is produced by the phosphorylation of glucose by HK-II, inhibits the interaction of HK-II and mTORC1. Moreover, a glucose deficiency in cardiomyocytes might enhance HK-II-mediated mTORC1 inhibition, which contributes to increased autophagy for supplying energy to protect cardiomyocytes (Roberts et al., 2014; Roberts and Miyamoto, 2015; Tan and Miyamoto, 2015). Tan et al. (2019) also confirmed that HK-II in mitochondria induces Parkin-mediated mitophagy to protect against myocardial ischemia. Similarly, in glucose-deprived cardiomyocytes, NADPH oxidase 4 (NOX4) produces reactive oxygen species and activates the protein kinase RNA-activated-like ER kinase signaling pathway to enhance autophagy and subsequent increase cardiomyocyte survival (Sciarretta et al., 2013). Taken together, during myocardial infarction, a deficiency in glucose metabolism substrates in cardiomyocytes enhances autophagy, which is an adaptive mechanism of the heart in response to ischemic stress that exerts a protective effectby replenishing energy and clearing damaged mitochondria (Figure 3). In contrast, other studies found that insulin-like growth factor 1 (IGF-1) inhibits autophagy in energy-deprived cardiomyocytes by activating protein kinase B (AKT)/mTOR and reducing AMPK activity, which activates mitochondrial metabolism, decreases cell death, and reduces the damage caused by excessive energy deprivation (Troncoso et al., 2012) (Figure 3). Therefore, we inferred that some potential mechanisms might determine whether autophagy induced by energy deprivation in cardiomyocytes promotes cardiomyocyte survival or increases cardiomyocyte death. High levels of autophagy potentially induce autosis, which directly leads to cardiomyocyte death. A threshold of autophagy flux may distinguish autosis from nonlethal autophagy (Kriel and Loos, 2019). A reasonable hypothesis is that this threshold may be one of the key mechanisms that determines cardiomyocyte death during myocardial infarction, but this hypothesis still requires further verification.

A shift from fatty acid to glucose metabolism caused by hypoxia has been observed in the hearts of mice with acute myocardial infarction (Doenst et al., 2013; Gu et al., 2018). Compared with the control group, mice that underwent three weeks of swimming training before the myocardial infarction showed a significant reduction in the infarct area. Mechanically, preoperative training improved mitochondrial biosynthesis and abrogated the change in energy metabolism by upregulating the expression of peroxisome proliferator-activated receptor-gamma coactivator-1 (PGC-1γ), which helped attenuate myocardial ischemia-induced autophagy (Tao et al., 2015). Therefore, an improvement in energy metabolism may limit autophagy to an appropriate level, which may represent an effective approach to ameliorating cardiac remodeling after myocardial infarction (Figure 3).

In contrast to the inhibition of mTOR by AMPK during myocardial ischemia, mTOR is activated during myocardial reperfusion through the inhibition of the glycogen synthase kinase-3β (GSK-3β)/tuberous sclerosis complex 2 (TCS2) pathway, resulting in decreased autophagy (Zhai et al., 2011; Sciarretta et al., 2014). Both melatonin and choline limit excess autophagy by activating mTOR, which ultimately reduces the infarct area after myocardial ischemia-reperfusion (Chen et al., 2018; Hang et al., 2018). Therefore, researchers have speculated that changes in energy metabolism due to reperfusion may result in a different effect of mTOR on protecting the heart from ischemia-reperfusion injury. Activated protein C (APC) reduces myocardial ischemia-reperfusion injury by increasing the oxidation of glucose rather than fatty acids as energy substrates and increasing autophagy flux (Costa et al., 2012). However, the systemic blockade of activin 2B receptor ligands protects against myocardial ischemia-reperfusion injury by increasing glucose uptake, increasing glycolysis and reducing autophagy (Magga et al., 2019). According to the aforementioned studies, increase in glucose metabolism appears to be beneficial for cardiac remodeling after ischemia-reperfusion injury. Nevertheless, this protective effect on myocardial ischemia-reperfusion injury was accompanied by different changes in autophagy in different experiments, increasing the difficulty of determining the exact relationship between energy homeostasis and autophagy. Because the degree of ischemia in different experiments may also affect the outcome of reperfusion, the effect of energy homeostasis on autophagy must be to clarified in further studies.

## Conclusion

In summary, autophagy regulates cardiac energy homeostasis during cardiac remodeling by releasing energy metabolism substrates and degrading key enzymes involved in energy metabolism, functioning as a “double-edged sword.” On the one hand, autophagy reduces oxidative stress and increases the survival of cardiomyocytes by scavenging dysfunctional mitochondria and replenishing the energysupply. On the other hand, autophagy aggravates pathological cardiac remodeling by increasing lipid deposition and reducing the efficiency of energy metabolism (Table 1). However, the mechanisms regulating autophagy and energy metabolism remain to be solved. Although many unsolved problems regarding the mechanism underlying the interaction between autophagy and energy homeostasis persist, the intracellular nutrient sensor mTOR plays a major role in this interaction during cardiac remodeling (Yang and Ming, 2012; Sciarretta et al., 2014; Tan and Miyamoto, 2016). Considering the pivotal functions of autophagy and energy homeostasis in the pathological mechanism of cardiac remodeling and the complex relationship between these processes, studies examining key mechanisms related to the interaction between autophagy and energy homeostasis may contribute to the discovery of new strategies to prevent or treat cardiac remodeling in the future.

**TABLE 1:** Autophagy and energy homeostasis in cardiovascular diseases.

Source	Model	Cardiovascular disease	Interaction between autophagy and energy homeostasis
Qin et al. (2017)	Endothelin-1-stimulated cardiomyocytes	Cardiac hypertrophy	Decreased intracellular amino acids increased autophagy-related proteins
Kim et al. (2014)	Mice with deficient amino acid sensor	Cardiac hypertrophy	The deficiency in amino acid sensor enhanced autophagy
Marino et al. (2014)	Mice with surgical thoracic aortic constriction	Cardiac hypertrophy	High levels of acetyl-CoA inhibited excessive autophagy
Shende et al. (2011)	Mice with deficient mammalian target of rapamycin complex 1	Cardiac hypertrophy	Enhanced autophagy reduced palmitate oxidation and increased glucose oxidation
Chen et al. (2017)	Mice with type 2 diabetes mellitus	Diabetes-associated cardiomyopathy	Increased fatty acids upregulated BECN1
Jaishy et al. (2015)	High-fat diet-fed mice	Diabetes-associated cardiomyopathy	Excess fatty acids blocked autophagy flux
An et al. (2017)	High-fat diet-fed mice	Diabetes-associated cardiomyopathy	Decreased autophagy increased the deposition of fatty acids
Tong et al. (2019)	High-fat diet-fed mice with autophagy-related 7 gene knockout	Diabetes-associated cardiomyopathy	Decreased autophagy increased lipid deposition
Zhang et al. (2016)	Mice with type 1 diabetes mellitus	Diabetes-associated cardiomyopathy	Lipid-lowering drug upregulated sirtuin1-mediated autophagy
Bockus et al. (2017)	Streptozotocin-treated diabetic mice and high-fat diet-fed mice	Diabetes-associated cardiomyopathy	Chaperone-mediated autophagy reduced energy metabolism efficiency
Tan et al. (2019)	Myocardial infarction mice	Ischemic heart diseases	Hexokinase-II induced mitophagy protected against myocardial infarction
Sciarretta et al. (2013)	Glucose-deprived cardiomyocytes	Ischemic heart diseases	Energy deprivation enhanced autophagy
Troncoso et al. (2012)	Energy-deprived cardiomyocytes	Ischemic heart diseases	Inhibited autophagy protected against energy deprivation in cardiomyocytes
Tao et al. (2015)	Myocardial infarction mice	Ischemic heart diseases	Improved energy metabolism attenuated ischemia-induced autophagy

## Author Contributions

WD and HF were responsible for assembling and drafting of the manuscript. W-J L was responsible for drawing figures and organizing tables. H-H L and Q-Z T were responsible for designing, conducting and revising this manuscript.

## Funding

This work was supported by the National Natural Science Foundation of China (No. 8180021781530012), the National Key R&D Program of China (No. 2018YFC1311300), the Development Center for Medical Science and Technology National Health and Family Planning Commission of the People’s Republic of China (The prevention and control project of cardiovascular disease, No. 2016ZX-008-01), the Fundamental Research Funds for the Central Universities (No. 2042018kf1032) and the Science and Technology Planning Projects of Wuhan (No. 2018061005132295).

## Conflict of Interest

The authors declare that the research was conducted in the absence of any commercial or financial relationships that could be construed as a potential conflict of interest.

## Abbreviations

mTORC1, mammalian target of rapamycin complex 1; mTOR, mammalian target of rapamycin; ULK1, unc-51 like kinase-1; LC3 I, microtubule-associated protein light chain I; LC3 II, microtubule-associated protein light chain II; p62, sequestosome 1; ACR, autophagy-related circular RNA; Atg5, autophagy-related 5; ACC2, acetyl-CoA-carboxylase 2; ACSLs, acyl-CoA synthetases; T2DM, type 2 diabetes mellitus; PKCβ, protein kinase C-β; NOX2, NADPH oxidase 2; LPL, lipoprotein lipase; ATG7, autophagy-related 7; T1DM, type 1 diabetes mellitus; PFK-2, phosphofructokinase 2; PFK-1, phosphofructokinase 1; HK-II, hexokinase-II; NOX4, NADPH oxidase 4; IGF-1, insulin-like growth factor 1; AKT, protein kinase B; PGC-1γ, peroxisome proliferator-activated receptor-gamma coactivator-1; GSK-3β, glycogen synthase kinase-3β; TCS2, tuberous sclerosis complex 2; APC, activated protein C.

## References

[B1] An M. Ryu D. R. Won Park J. Ha Choi J. Park E. M. Eun Lee K. (2017). ULK1 prevents cardiac dysfunction in obesity through autophagy-meditated regulation of lipid metabolism. Cardiovasc. Res. 113 (10), 1137–1147. 10.1093/cvr/cvx064 28430962

[B2] Anderson E. J. Kypson A. P. Rodriguez E. Anderson C. A. Lehr E. J. Neufer P. D. (2009). Substrate-specific derangements in mitochondrial metabolism and redox balance in the atrium of the type 2 diabetic human heart. J. Am. Coll. Cardiol. 54 (20), 1891–1898. 10.1016/j.jacc.2009.07.031 19892241 PMC2800130

[B3] Anding A. L. Baehrecke E. H. (2017). Cleaning house: selective autophagy of organelles. Dev. Cell 41 (1), 10–22. 10.1016/j.devcel.2017.02.016 28399394 PMC5395098

[B4] Ashrafian H. Frenneaux M. P. Opie L. H. (2007). Metabolic mechanisms in heart failure. Circulation 116 (4), 434–448. 10.1161/CIRCULATIONAHA.107.702795 17646594

[B5] Bar-Peled L. Sabatini D. M. (2014). Regulation of mTORC1 by amino acids. Trends Cell Biol. 24 (7), 400–406. 10.1016/j.tcb.2014.03.003 24698685 PMC4074565

[B6] Bedi K. C. Jr. Snyder N. W. Brandimarto J. Aziz M. Mesaros C. Worth A. J. (2016). Evidence for intramyocardial disruption of lipid metabolism and increased myocardial ketone utilization in advanced human heart failure. Circulation 133 (8), 706–716. 10.1161/CIRCULATIONAHA.115.017545 26819374 PMC4779339

[B7] Bertero E. Maack C. (2018). Metabolic remodelling in heart failure. Nat. Rev. Cardiol. 15 (8), 457–470. 10.1038/s41569-018-0044-6 29915254

[B8] Bockus L. B. Matsuzaki S. Vadvalkar S. S. Young Z. T. Giorgione J. R. Newhardt M. F. (2017). Cardiac insulin signaling regulates glycolysis through phosphofructokinase 2 content and activity. J. Am. Heart Assoc. 6 (12). 10.1161/JAHA.117.007159 PMC5779029 29203581

[B9] Boudina S. Sena S. Theobald H. Sheng X. Wright J. J. Hu X. X. (2007). Mitochondrial energetics in the heart in obesity-related diabetes: direct evidence for increased uncoupled respiration and activation of uncoupling proteins. Diabetes 56 (10), 2457–2466. 10.2337/db07-0481 17623815

[B10] Buss S. J. Muenz S. Riffel J. H. Malekar P. Hagenmueller M. Weiss C. S. (2009). Beneficial effects of Mammalian target of rapamycin inhibition on left ventricular remodeling after myocardial infarction. J. Am. Coll. Cardiol. 54 (25), 2435–2446. 10.1016/j.jacc.2009.08.031 20082935

[B11] Chen C. Yang S. Li H. Yin Z. Fan J. Zhao Y. (2017). Mir30c is involved in diabetic cardiomyopathy through regulation of cardiac autophagy via BECN1. Mol. Ther. Nucleic Acids 7, 127–139. 10.1016/j.omtn.2017.03.005 28624189 PMC5415963

[B12] Chen W. R. Liu H. B. Chen Y. D. Sha Y. Ma Q. Zhu P. J. (2018). Melatonin attenuates myocardial ischemia/reperfusion injury by inhibiting autophagy via an AMPK/mTOR signaling pathway. Cell. Physiol. Biochem. 47 (5), 2067–2076. 10.1159/000491474 29975938

[B13] Costa R. Morrison A. Wang J. Manithody C. Li J. Rezaie A. R. (2012). Activated protein C modulates cardiac metabolism and augments autophagy in the ischemic heart. J. Thromb. Haemost. 10 (9), 1736–1744. 10.1111/j.1538-7836.2012.04833.x 22738025 PMC3433592

[B14] Delbridge L. M. D. Mellor K. M. Taylor D. J. Gottlieb R. A. (2017). Myocardial stress and autophagy: mechanisms and potential therapies. Nat. Rev. Cardiol. 14 (7), 412–425. 10.1038/nrcardio.2017.35 28361977 PMC6245608

[B15] Dikic I. Elazar Z. (2018). Mechanism and medical implications of mammalian autophagy. Nat. Rev. Mol. Cell Biol. 19 (6), 349–364. 10.1038/s41580-018-0003-4 29618831

[B16] Doenst T. Nguyen T. D. Abel E. D. (2013). Cardiac metabolism in heart failure: implications beyond ATP production. Circ. Res. 113 (6), 709–724. 10.1161/CIRCRESAHA.113.300376 23989714 PMC3896379

[B17] Doenst T. Pytel G. Schrepper A. Amorim P. Farber G. Shingu Y. (2010). Decreased rates of substrate oxidation *ex vivo* predict the onset of heart failure and contractile dysfunction in rats with pressure overload. Cardiovasc. Res. 86 (3), 461–470. 10.1093/cvr/cvp414 20035032

[B18] Dolinsky V. W. Chan A. Y. Robillard Frayne I. Light P. E. Des Rosiers C. Dyck J. R. (2009). Resveratrol prevents the prohypertrophic effects of oxidative stress on LKB1. Circulation 119 (12), 1643–1652. 10.1161/CIRCULATIONAHA.108.78744 19289642

[B19] Dorn G. W. 2nd (2019). Evolving concepts of mitochondrial dynamics. Annu. Rev. Physiol. 81, 1–17. 10.1146/annurev-physiol-020518-114358 30256725

[B20] Florey O. Overholtzer M. (2012). Autophagy proteins in macroendocytic engulfment. Trends Cell Biol. 22 (7), 374–380. 10.1016/j.tcb.2012.04.005 22608991 PMC3383932

[B21] Fu Y. N. Xiao H. Ma X. W. Jiang S. Y. Xu M. Zhang Y. Y. (2011). Metformin attenuates pressure overload-induced cardiac hypertrophy via AMPK activation. Acta Pharmacol. Sin. 32 (7), 879–887. 10.1038/aps.2010.229 21552292 PMC4003117

[B22] Galluzzi L. Pietrocola F. Levine B. Kroemer G. (2014). Metabolic control of autophagy. Cell 159 (6), 1263–1276. 10.1016/j.cell.2014.11.006 25480292 PMC4500936

[B23] Gibb A. A. Hill B. G. (2018). Metabolic coordination of physiological and pathological cardiac remodeling. Circ. Res. 123 (1), 107–128. 10.1161/CIRCRESAHA.118.312017 29929976 PMC6023588

[B24] Grevengoed T. J. Cooper D. E. Young P. A. Ellis J. M. Coleman R. A. (2015). Loss of long-chain acyl-CoA synthetase isoform 1 impairs cardiac autophagy and mitochondrial structure through mechanistic target of rapamycin complex 1 activation. FASEB J 29 (11), 4641–4653. 10.1096/fj.15-272732 26220174 PMC4608904

[B25] Gu C. Li T. Jiang S. Yang Z. Lv J. Yi W. (2018). AMP-activated protein kinase sparks the fire of cardioprotection against myocardial ischemia and cardiac ageing. Ageing Res. Rev. 47, 168–175. 10.1016/j.arr.2018.08.002 30110651

[B26] Gustafsson A. B. Gottlieb R. A. (2008). Recycle or die: the role of autophagy in cardioprotection. J. Mol. Cell. Cardiol. 44 (4), 654–661. 10.1016/j.yjmcc.2008.01.010 18353358 PMC2423346

[B27] Gustafsson A. B. Gottlieb R. A. (2009). Autophagy in ischemic heart disease. Circ. Res. 104 (2), 150–158. 10.1161/CIRCRESAHA.108.187427 19179668 PMC2765251

[B28] Hamacher-Brady A. (2012). Autophagyregulation and integration with cell signaling. Antioxidants Redox Signal. 17 (5), 756–765. 10.1089/ars.2011.4410 22149388

[B29] Hang P. Zhao J. Su Z. Sun H. Chen T. Zhao L. (2018). Choline inhibits ischemia-reperfusion-induced cardiomyocyte autophagy in rat myocardium by activating akt/mTOR signaling. Cell. Physiol. Biochem. 45 (5), 2136–2144. 10.1159/000488049 29533930

[B30] Hariharan N. Maejima Y. Nakae J. Paik J. Depinho R. A. Sadoshima J. (2010). Deacetylation of FoxO by Sirt1 plays an essential role in mediating starvation-induced autophagy in cardiac myocytes. Circ. Res. 107 (12), 1470–1482. 10.1161/CIRCRESAHA.110.227371 20947830 PMC3011986

[B31] He C. Bassik M. C. Moresi V. Sun K. Wei Y. Zou Z. (2012). Exercise-induced BCL2-regulated autophagy is required for muscle glucose homeostasis. Nature 481 (7382), 511–515. 10.1038/nature10758 22258505 PMC3518436

[B32] Ingwall J. S. (2009). Energy metabolism in heart failure and remodelling. Cardiovasc. Res. 81 (3), 412–419. 10.1093/cvr/cvn301 18987051 PMC2639129

[B33] Jaishy B. Zhang Q. Chung H. S. Riehle C. Soto J. Jenkins S. (2015). Lipid-induced NOX2 activation inhibits autophagic flux by impairing lysosomal enzyme activity. J. Lipid Res. 56 (3), 546–561. 10.1194/jlr.M055152 25529920 PMC4340303

[B34] Jewell J. L. Russell R. C. Guan K. L. (2013). Amino acid signalling upstream of mTOR. Nat. Rev. Mol. Cell Biol. 14 (3), 133–139. 10.1038/nrm3522 23361334 PMC3988467

[B35] Johansen T. Lamark T. (2011). Selective autophagy mediated by autophagic adapter proteins. Autophagy 7 (3), 279–296. 10.4161/auto.7.3.14487 21189453 PMC3060413

[B36] Kanamori H. Takemura G. Goto K. Maruyama R. Tsujimoto A. Ogino A. (2011). The role of autophagy emerging in postinfarction cardiac remodelling. Cardiovasc. Res. 91 (2), 330–339. 10.1093/cvr/cvr073 21406597

[B37] Kanamori H. Takemura G. Goto K. Tsujimoto A. Mikami A. Ogino A. (2015). Autophagic adaptations in diabetic cardiomyopathy differ between type 1 and type 2 diabetes. Autophagy 11 (7), 1146–1160. 10.1080/15548627.2015.1051295 26042865 PMC4590644

[B38] Kemp B. E. Mitchelhill K. I. Stapleton D. Michell B. J. Chen Z. P. Witters L. A. (1999). Dealing with energy demand: the AMP-activated protein kinase. Trends Biochem. Sci. 24 (1), 22–25. 10.1016/s0968-0004(98)01340-1 10087918

[B39] Kim Y. C. Park H. W. Sciarretta S. Mo J. S. Jewell J. L. Russell R. C. (2014). Rag GTPases are cardioprotective by regulating lysosomal function. Nat. Commun. 5, 4241 10.1038/ncomms5241 24980141 PMC4100214

[B40] Kolwicz S. C. Jr. Purohit S. Tian R. (2013). Cardiac metabolism and its interactions with contraction, growth, and survival of cardiomyocytes. Circ. Res. 113 (5), 603–616. 10.1161/CIRCRESAHA.113.302095 23948585 PMC3845521

[B41] Kriel J. Loos B. (2019). The good, the bad and the autophagosome: exploring unanswered questions of autophagy-dependent cell death. Cell Death Differ. 26 (4), 640–652. 10.1038/s41418-018-0267-4 30659234 PMC6460391

[B42] Krishnan J. Suter M. Windak R. Krebs T. Felley A. Montessuit C. (2009). Activation of a HIF1alpha-PPARgamma axis underlies the integration of glycolytic and lipid anabolic pathways in pathologic cardiac hypertrophy. Cell Metabol. 9 (6), 512–524. 10.1016/j.cmet.2009.05.005 19490906

[B43] Lopaschuk G. D. Ussher J. R. Folmes C. D. Jaswal J. S. Stanley W. C. (2010). Myocardial fatty acid metabolism in health and disease. Physiol. Rev. 90 (1), 207–258. 10.1152/physrev.00015.2009 20086077

[B44] Ma X. Liu H. Foyil S. R. Godar R. J. Weinheimer C. J. Hill J. A. (2012). Impaired autophagosome clearance contributes to cardiomyocyte death in ischemia/reperfusion injury. Circulation 125 (25)**v** 3170–3181. 10.1161/CIRCULATIONAHA.111.041814 22592897 PMC3397471

[B45] Magga J. Vainio L. Kilpio T. Hulmi J. J. Taponen S. Lin R. (2019). Systemic blockade of ACVR2B ligands protects myocardium from acute ischemia-reperfusion injury. Mol. Ther. 27 (3), 600–610. 10.1016/j.ymthe.2019.01.013 30765322 PMC6404100

[B46] Marino G. Pietrocola F. Eisenberg T. Kong Y. Malik S. A. Andryushkova A. (2014). Regulation of autophagy by cytosolic acetyl-coenzyme A. Mol. Cell 53 (5), 710–725. 10.1016/j.molcel.2014.01.016 24560926

[B47] Matsui Y. Takagi H. Qu X. Abdellatif M. Sakoda H. Asano T. (2007). Distinct roles of autophagy in the heart during ischemia and reperfusion: roles of AMP-activated protein kinase and Beclin 1 in mediating autophagy. Circ. Res. 100 (6), 914–922. 10.1161/01.RES.0000261924.76669.36 17332429

[B48] Miyamoto S. (2019). Autophagy and cardiac aging. Cell Death Differ. 26 (4), 653–664. 10.1038/s41418-019-0286-9 30692640 PMC6460392

[B49] Nah J. Zhai P. Huang C. Y. Fernandez A. F. Mareedu S. Levine B. (2020). Upregulation of Rubicon promotes autosis during myocardial ischemia/reperfusion injury. J. Clin. Invest. 130 (6), 2978–2991. 10.1172/JCI132366 32364533 PMC7260042

[B50] Nakai A. Yamaguchi O. Takeda T. Higuchi Y. Hikoso S. Taniike M. (2007). The role of autophagy in cardiomyocytes in the basal state and in response to hemodynamic stress. Nat. Med. 13 (5), 619–624. 10.1038/nm1574 17450150

[B51] Nakamura M. Sadoshima J. (2018). Mechanisms of physiological and pathological cardiac hypertrophy. Nat. Rev. Cardiol. 15 (7), 387–407. 10.1038/s41569-018-0007-y 29674714

[B52] Noordali H. Loudon B. L. Frenneaux M. P. Madhani M. (2018). Cardiac metabolism - a promising therapeutic target for heart failure. Pharmacol. Ther. 182, 95–114. 10.1016/j.pharmthera.2017.08.001 28821397

[B53] Qi D. Young L. H. (2015). AMPK: energy sensor and survival mechanism in the ischemic heart. Trends Endocrinol. Metab. 26 (8), 422–429. 10.1016/j.tem.2015.05.010 26160707 PMC4697457

[B54] Qin P. Arabacilar P. Bernard R. E. Bao W. Olzinski A. R. Guo Y. (2017). Activation of the amino acid response pathway blunts the effects of cardiac stress. J. Am. Heart Assoc. 6 (5). 10.1161/JAHA.116.004453 PMC5524058 28487390

[B55] Ritterhoff J. Young S. Villet O. Shao D. Neto F. C. Bettcher L. F. (2020). Metabolic remodeling promotes cardiac hypertrophy by directing glucose to aspartate biosynthesis. Circ. Res. 126 (2), 182–196. 10.1161/CIRCRESAHA.119.315483 31709908 PMC8448129

[B56] Roberts D. J. Miyamoto S. (2015). Hexokinase II integrates energy metabolism and cellular protection: akting on mitochondria and TORCing to autophagy. Cell Death Differ. 22 (2), 248–257. 10.1038/cdd.2014.173 25323588 PMC4291497

[B57] Roberts D. J. Tan-Sah V. P. Smith J. E. Miyamoto S. (2014). Hexokinase-II positively regulates glucose starvation-induced autophagy through TORC1 inhibition. Mol. Cell 53 (4), 521–533. 10.1016/j.molcel.2013.12.019 24462113 PMC3943874

[B58] Rothermel B. A. Hill J. A. (2008). Autophagy in load-induced heart disease. Circ. Res. 103 (12), 1363–1369. 10.1161/CIRCRESAHA.108.186551 19059838 PMC2607044

[B59] Santos-Gallego C. G. Requena-Ibanez J. A. San Antonio R. Ishikawa K. Watanabe S. Picatoste B. (2019). Empagliflozin ameliorates adverse left ventricular remodeling in nondiabetic heart failure by enhancing myocardial energetics. J. Am. Coll. Cardiol. 73 (15), 1931–1944. 10.1016/j.jacc.2019.01.056 30999996

[B60] Sciarretta S. Maejima Y. Zablocki D. Sadoshima J. (2018a). The role of autophagy in the heart. Annu. Rev. Physiol. 80, 1–26. 10.1146/annurev-physiol-021317-121427 29068766

[B61] Sciarretta S. Volpe M. Sadoshima J. (2014). Mammalian target of rapamycin signaling in cardiac physiology and disease. Circ. Res. 114 (3), 549–564. 10.1161/CIRCRESAHA.114.302022 24481845 PMC3995130

[B62] Sciarretta S. Yee D. Nagarajan N. Bianchi F. Saito T. Valenti V. (2018b). Trehalose-induced activation of autophagy improves cardiac remodeling after myocardial infarction. J. Am. Coll. Cardiol. 71 (18), 1999–2010. 10.1016/j.jacc.2018.02.066 29724354 PMC6347412

[B63] Sciarretta S. Zhai P. Shao D. Maejima Y. Robbins J. Volpe M. (2012). Rheb is a critical regulator of autophagy during myocardial ischemia: pathophysiological implications in obesity and metabolic syndrome. Circulation 125 (9), 1134–1146. 10.1161/CIRCULATIONAHA.111.078212 22294621 PMC3337789

[B64] Sciarretta S. Zhai P. Shao D. Zablocki D. Nagarajan N. Terada L. S. (2013). Activation of NADPH oxidase 4 in the endoplasmic reticulum promotes cardiomyocyte autophagy and survival during energy stress through the protein kinase RNA-activated-like endoplasmic reticulum kinase/eukaryotic initiation factor 2alpha/activating transcription factor 4 pathway. Circ. Res. 113 (11), 1253–1264. 10.1161/CIRCRESAHA.113.301787 24081881 PMC3937770

[B65] Shende P. Plaisance I. Morandi C. Pellieux C. Berthonneche C. Zorzato F. (2011). Cardiac raptor ablation impairs adaptive hypertrophy, alters metabolic gene expression, and causes heart failure in mice. Circulation 123 (10), 1073–1082. 10.1161/CIRCULATIONAHA.110.977066 21357822

[B66] Shi B. Ma M. Zheng Y. Pan Y. Lin X. (2019). mTOR and Beclin1: two key autophagy-related molecules and their roles in myocardial ischemia/reperfusion injury. J. Cell. Physiol. 234 (8), 12562–12568. 10.1002/jcp.28125 30618070 PMC13484135

[B67] Shimizu I. Minamino T. (2016). Physiological and pathological cardiac hypertrophy. J. Mol. Cell. Cardiol. 97, 245–262. 10.1016/j.yjmcc.2016.06.001 27262674

[B68] Sorokina N. O'Donnell J. M. McKinney R. D. Pound K. M. Woldegiorgis G. LaNoue K. F. (2007). Recruitment of compensatory pathways to sustain oxidative flux with reduced carnitine palmitoyltransferase I activity characterizes inefficiency in energy metabolism in hypertrophied hearts. Circulation 115 (15), 2033–2041. 10.1161/CIRCULATIONAHA.106.668665 17404155

[B69] Stanley W. C. Recchia F. A. Lopaschuk G. D. (2005). Myocardial substrate metabolism in the normal and failing heart. Physiol. Rev. 85 (3), 1093–1129. 10.1152/physrev.00006.2004 15987803

[B70] Takagi H. Matsui Y. Hirotani S. Sakoda H. Asano T. Sadoshima J. (2007). AMPK mediates autophagy during myocardial ischemia *in vivo* . Autophagy 3 (4), 405–407. 10.4161/auto.4281 17471015

[B71] Takemura G. Kanamori H. Goto K. Maruyama R. Tsujimoto A. Fujiwara H. (2009). Autophagy maintains cardiac function in the starved adult. Autophagy 5 (7), 1034–1036. 10.4161/auto.5.7.9297 19587530

[B72] Tan V. P. Miyamoto S. (2015). HK2/hexokinase-II integrates glycolysis and autophagy to confer cellular protection. Autophagy 11 (6), 963–964. 10.1080/15548627.2015.1042195 26075878 PMC4502787

[B73] Tan V. P. Miyamoto S. (2016). Nutrient-sensing mTORC1: integration of metabolic and autophagic signals. J. Mol. Cell. Cardiol. 95, 31–41. 10.1016/j.yjmcc.2016.01.005 26773603 PMC4909545

[B74] Tan V. P. Smith J. M. Tu M. Yu J. D. Ding E. Y. Miyamoto S. (2019). Dissociation of mitochondrial HK-II elicits mitophagy and confers cardioprotection against ischemia. Cell Death Dis. 10 (10), 730 10.1038/s41419-019-1965-7 31570704 PMC6768853

[B75] Tan Y. Zhang Z. Zheng C. Wintergerst K. A. Keller B. B. Cai L. (2020). Mechanisms of diabetic cardiomyopathy and potential therapeutic strategies: preclinical and clinical evidence. Nat. Rev. Cardiol. 17, 585–607. 10.1038/s41569-020-0339-2 32080423 PMC7849055

[B76] Tao L. Bei Y. Lin S. Zhang H. Zhou Y. Jiang J. (2015). Exercise training protects against acute myocardial infarction via improving myocardial energy metabolism and mitochondrial biogenesis. Cell. Physiol. Biochem. 37 (1), 162–175. 10.1159/000430342 26303678

[B77] Tham Y. K. Bernardo B. C. Ooi J. Y. Weeks K. L. McMullen J. R. (2015). Pathophysiology of cardiac hypertrophy and heart failure: signaling pathways and novel therapeutic targets. Arch. Toxicol. 89 (9), 1401–1438. 10.1007/s00204-015-1477-x 25708889

[B78] Tong M. Saito T. Zhai P. Oka S. I. Mizushima W. Nakamura M. (2019). Mitophagy is essential for maintaining cardiac function during high fat diet-induced diabetic cardiomyopathy. Circ. Res. 124 (9), 1360–1371. 10.1161/CIRCRESAHA.118.314607 30786833 PMC6483841

[B79] Troncoso R. Vicencio J. M. Parra V. Nemchenko A. Kawashima Y. Del Campo A. (2012). Energy-preserving effects of IGF-1 antagonize starvation-induced cardiac autophagy. Cardiovasc. Res. 93 (2), 320–329. 10.1093/cvr/cvr321 22135164 PMC3286200

[B80] Tuunanen H. Engblom E. Naum A. Nagren K. Hesse B. Airaksinen K. E. (2006). Free fatty acid depletion acutely decreases cardiac work and efficiency in cardiomyopathic heart failure. Circulation 114 (20), 2130–2137. 10.1161/CIRCULATIONAHA.106.645184 17088453

[B81] Tuunanen H. Knuuti J. (2011). Metabolic remodelling in human heart failure. Cardiovasc. Res. 90 (2), 251–257. 10.1093/cvr/cvr052 21372005

[B82] Vigetti D. Caon I. Passi A. (2018). A nutrient sentinel stands guard outside the cell. J. Biol. Chem. 293 (43), 16951–16952. 10.1074/jbc.H118.006101 30366972 PMC6204894

[B83] Wang X. Guo Z. Ding Z. Mehta J. L. (2018). Inflammation, autophagy, and apoptosis after myocardial infarction. J. Am. Heart Assoc. 7 (9), e008024 10.1161/JAHA.117.008024 29680826 PMC6015297

[B84] Wauson E. M. Zaganjor E. Lee A. Y. Guerra M. L. Ghosh A. B. Bookout A. L. (2012). The G protein-coupled taste receptor T1R1/T1R3 regulates mTORC1 and autophagy. Mol. Cell 47 (6), 851–862. 10.1016/j.molcel.2012.08.001 22959271 PMC3749915

[B85] Wei H. Qu H. Wang H. Ji B. Ding Y. Liu D. (2017). 1,25-Dihydroxyvitamin-D3 prevents the development of diabetic cardiomyopathy in type 1 diabetic rats by enhancing autophagy via inhibiting the beta-catenin/TCF4/GSK-3beta/mTOR pathway. J. Steroid Biochem. Mol. Biol. 168, 71–90. 10.1016/j.jsbmb.2017.02.007 28216152

[B86] Wu Q. Q. Xiao Y. Yuan Y. Ma Z. G. Liao H. H. Liu C. (2017). Mechanisms contributing to cardiac remodelling. Clin. Sci. (Lond.) 131 (18), 2319–2345. 10.1042/CS20171167 28842527

[B87] Xie M. Kong Y. Tan W. May H. Battiprolu P. K. Pedrozo Z. (2014). Histone deacetylase inhibition blunts ischemia/reperfusion injury by inducing cardiomyocyte autophagy. Circulation 129 (10), 1139–1151. 10.1161/CIRCULATIONAHA.113.002416 24396039 PMC3984913

[B88] Xie Z. He C. Zou M. H. (2011a). AMP-activated protein kinase modulates cardiac autophagy in diabetic cardiomyopathy. Autophagy 7 (10), 1254–1255. 10.4161/auto.7.10.16740 21685727 PMC3210311

[B89] Xie Z. Lau K. Eby B. Lozano P. He C. Pennington B. (2011b). Improvement of cardiac functions by chronic metformin treatment is associated with enhanced cardiac autophagy in diabetic OVE26 mice. Diabetes 60 (6), 1770–1778. 10.2337/db10-0351 21562078 PMC3114402

[B90] Yang Z. Ming X. F. (2012). mTOR signalling: the molecular interface connecting metabolic stress, aging and cardiovascular diseases. Obes. Rev. 13 (Suppl. 2), 58–68. 10.1111/j.1467-789X.2012.01038.x 23107260

[B91] Zhai P. Sciarretta S. Galeotti J. Volpe M. Sadoshima J. (2011). Differential roles of GSK-3beta during myocardial ischemia and ischemia/reperfusion. Circ. Res. 109 (5), 502–511. 10.1161/CIRCRESAHA.111.249532 21737790 PMC3158807

[B92] Zhang J. Cheng Y. Gu J. Wang S. Zhou S. Wang Y. (2016). Fenofibrate increases cardiac autophagy via FGF21/SIRT1 and prevents fibrosis and inflammation in the hearts of Type 1 diabetic mice. Clin. Sci. (Lond.) 130 (8), 625–641. 10.1042/CS20150623 26795437

[B93] Zhang Y. Wang Y. Xu J. Tian F. Hu S. Chen Y. (2019). Melatonin attenuates myocardial ischemia-reperfusion injury via improving mitochondrial fusion/mitophagy and activating the AMPK-OPA1 signaling pathways. J. Pineal Res. 66 (2), e12542 10.1111/jpi.12542 30516280

[B94] Zhou L. Y. Zhai M. Huang Y. Xu S. An T. Wang Y. H. (2019). The circular RNA ACR attenuates myocardial ischemia/reperfusion injury by suppressing autophagy via modulation of the Pink1/FAM65B pathway. Cell Death Differ. 26 (7), 1299–1315. 10.1038/s41418-018-0206-4 30349076 PMC6748144

[B95] Zhu H. Tannous P. Johnstone J. L. Kong Y. Shelton J. M. Richardson J. A. (2007). Cardiac autophagy is a maladaptive response to hemodynamic stress. J. Clin. Invest. 117 (7), 1782–1793. 10.1172/JCI27523 17607355 PMC1890995

[B96] Zou M. H. Xie Z. (2013). Regulation of interplay between autophagy and apoptosis in the diabetic heart: new role of AMPK. Autophagy 9 (4), 624–625. 10.4161/auto.23577 23380689 PMC3627682

